# Potential reversal of epigenetic age using a diet and lifestyle intervention: a pilot randomized clinical trial

**DOI:** 10.18632/aging.202913

**Published:** 2021-04-12

**Authors:** Kara N. Fitzgerald, Romilly Hodges, Douglas Hanes, Emily Stack, David Cheishvili, Moshe Szyf, Janine Henkel, Melissa W. Twedt, Despina Giannopoulou, Josette Herdell, Sally Logan, Ryan Bradley

**Affiliations:** 1 Institute for Functional Medicine, Federal Way, WA 98003, USA; 2 American Nutrition Association, Hinsdale, IL 60521, USA; 3 Helfgott Research Institute, National University of Natural Medicine, Portland, OR 97201, USA; 4 Helfgott Research Institute, National University of Natural Medicine, Portland, OR 97201, USA; 5 HKG Epitherapeutics (Hong Kong), Department of Molecular Biology, Ariel University, Israel, Gerald Bronfman Department of Oncology, McGill University, Montreal, Quebec, Canada; 6 Department of Pharmacology and Therapeutics, McGill University, Montreal, QC H3G 1Y6, Canada; 7 Helfgott Research Institute, National University of Natural Medicine, Portland, OR 97201, USA; 8 Division of Preventive Medicine, University of California, San Diego, CA 92023, USA

**Keywords:** DNA methylation, epigenetic, aging, lifestyle, biological clock

## Abstract

Manipulations to slow biological aging and extend healthspan are of interest given the societal and healthcare costs of our aging population. Herein we report on a randomized controlled clinical trial conducted among 43 healthy adult males between the ages of 50-72. The 8-week treatment program included diet, sleep, exercise and relaxation guidance, and supplemental probiotics and phytonutrients. The control group received no intervention. Genome-wide DNA methylation analysis was conducted on saliva samples using the Illumina Methylation Epic Array and DNAmAge was calculated using the online Horvath DNAmAge clock (2013). The diet and lifestyle treatment was associated with a 3.23 years decrease in DNAmAge compared with controls (p=0.018). DNAmAge of those in the treatment group decreased by an average 1.96 years by the end of the program compared to the same individuals at the beginning with a strong trend towards significance (p=0.066). Changes in blood biomarkers were significant for mean serum 5-methyltetrahydrofolate (+15%, p=0.004) and mean triglycerides (-25%, p=0.009). To our knowledge, this is the first randomized controlled study to suggest that specific diet and lifestyle interventions may reverse Horvath DNAmAge (2013) epigenetic aging in healthy adult males. Larger-scale and longer duration clinical trials are needed to confirm these findings, as well as investigation in other human populations.

## INTRODUCTION

Advanced age is the largest risk factor for impaired mental and physical function and many non-communicable diseases including cancer, neurodegeneration, type 2 diabetes, and cardiovascular disease [1, 2]. The growing health-related economic and social challenges of our rapidly aging population are well recognized and affect individuals, their families, health systems and economies. Considering economics alone, delaying aging by 2.2 years (with associated extension of healthspan) could save $7 trillion over fifty years [3]. This broad approach was identified to be a much better investment than disease-specific spending. Thus, if interventions can be identified that extend healthspan even modestly, benefits for public health and healthcare economics will be substantial.

DNA methylation is the addition of a methyl group to cytosine residues at selective areas on a chromosome (e.g. CpG islands, shelf/shore, exons, open sea). Methylation constitutes the best-studied, and likely most resilient of many mechanisms controlling gene expression [4]. Unique among epigenetic markers, DNA methylation can readily and cheaply be mapped from tissue samples. Of 20+ million methylation sites on the human genome, there are a few thousand at which methylation levels are tightly correlated with age. Currently, the best biochemical markers of an individual’s age are all based on patterns of methylation [5]. This has led some researchers to propose that aging itself has its basis in epigenetic changes (including methylation changes) over time [6–9].

As of this writing, the best-studied methylation-based clock is the multi-tissue DNAmAge clock [10]. At the time this study design was approved, there were few viable alternatives. Horvath’s DNAmAge clock predicts all-cause mortality and multiple morbidities better than chronological age. Methylation clocks (including DNAmAge) are based on systematic methylation changes with age. DNAmAge clock specifically demonstrates about 60% of CpG sites losing methylation with age and 40% gaining methylation. This is distinct from stochastic changes, “methylation drift”, unpredictable changes which vary among individuals and cell-by-cell within individuals. Systematic methylation changes include hypermethylation in promotor regions of tumor suppressor genes (inhibiting expression) and hypomethylation promoting inflammatory cytokines (promoting expression). Saliva can be considered a good source of high-quality DNA, containing both white blood cells and buccal cells, and is a suitable tissue type to be assessed for the DNAmAge clock [10, 11].

The dietary recommendations employed as part of the treatment protocol for this study were based largely on biochemistry and generalized measures of health, because few dietary associations with the DNAmAge clock have yet been established. A modest, but significant, reduction in DNAmAge in individuals consuming a non-specific lean meat, fish and plant-based diet (as measured by blood carotenoids) has been observed [12]. It is possible that changes of a greater magnitude require a more targeted approach. The dietary intervention used here was also plant-centered, but including a high intake of nutrients that are substrates or cofactors in methylation biosynthetic pathways (e.g. containing folate, betaine), ten-eleven translocation (TET) demethylase cofactors and modulators (e.g. alpha ketoglutarate, vitamin C and vitamin A) [13] and polyphenolic modulators of DNA methyl transferases (DNMT) (e.g. curcumin, epigallocatechin gallate (EGCG), rosmarinic acid, quercetin, luteolin) [14]. It also included limited nutrient-dense animal proteins (e.g. liver, egg). The diet restricted carbohydrates and included mild intermittent fasting, both designed to lower glycemic cycling. The diet was supplemented daily with a fruit and vegetable powder, also rich in polyphenolic modulators of DNMT activity, and a probiotic providing 40 million CFU of *Lactobacillus plantarum* 299v. *L. plantarum* has been shown to be a folate producer in the presence of para aminobenzoic acid (PABA) [15]; it also has been demonstrated to alter gene expression [16].

Lifestyle guidance in this study included a minimum of 30 minutes of exercise per day, at least 5 days per week at an intensity of 60-80 percent of maximum perceived exertion. Exercise is well-known to be broadly beneficial for almost every aspect of health and has been shown to extend mean lifespan in animal models. Exploration of the effect of exercise on the methylome has recently begun. For example, regular *tai chi* practice was associated with slowing of age-related DNA methylation losses in 500 women [17]. In another study of 647 women, a lifelong history of exercise was associated with a similar endpoint [18]. These results were not reported in terms of the Horvath clock, because it had not yet been developed. One systematic review of human studies found that regular, daily physical activity was associated with lower blood levels of homocysteine, which when elevated, suggests an insufficiency of methylation capacity [19]. Excessive exercise may accelerate methylation aging, but this danger has only been observed in elite, competitive athletes [20].

Twice-daily breathing exercises that elicit the Relaxation Response were prescribed for stress reduction. It was recently demonstrated that 60 days of relaxation practice designed to elicit the Relaxation Response, 20 minutes twice per day, could significantly reduce DNAmAge as measured by the Zbieć-Piekarska clock in their group of healthy participants (though not in their ‘patient’ group) [21]. *Almost* a quarter of the DNAmAge CpG sites (85/353) are located in glucocorticoid response elements, pointing to a likely relationship between stress and accelerated aging. Cumulative lifetime stress has been shown to be associated with accelerated aging of the methylome [22]. Zannas et al. also reported that dexamethasone, a glucocorticoid agonist, can advance the DNAmAge clock and induce associated transcriptional changes. Dexamethasone-regulated genes showed enriched association of aging-related diseases, including coronary artery disease, arteriosclerosis and leukemias. Other findings include that PTSD contributes to accelerated methylation age [23]; and that greater infant distress (lack of caregiver contact) is associated with an underdeveloped, younger epigenetic age [24].

This study aimed to optimize sleep, with a recommendation for at least seven hours nightly. Seven hours is generally considered to be healthy [25], but the limited data on accelerated aging only relates to extremes of sleep deprivation. A (presumably transient) effect of sleep deprivation on genome-wide methylation patterns in blood has been demonstrated [26]. Acceleration of the DNAmAge clock has been associated with insomnia in a sample of 2078 women [27]. Carskadon et al [28] found an association between poor quality / fewer hours of sleep with age acceleration in a small sample of 12 female college students.

This multimodal (“systems”) intervention is reflective of a clinically-used approach that combines individual interventions, each of which carry evidence of favorable influence on the DNA methylome and of which several authors of this study have clinical experience of health benefits. Such interventions likely produce synergistic effects and reduce the possibility of negative effects from one disease-promoting input canceling out the benefits of another health-promoting input. Dietary and lifestyle interventions, as used here, target upstream influences that are generally considered safe, even over the long term.

By design, an important endpoint of this study was to be Horvath’s DNAmAge clock, to see if it could be potentially slowed. This is to say we have tentatively accepted the hypothesis that the methylation pattern from which the DNAmAge clock is computed is a driver of aging (and the chronic diseases of aging), thus we expect that attempting to directly influence the DNA methylome using diet and lifestyle to set back DNAmAge will lead to a healthier, more “youthful” metabolism. To date, three non-controlled studies have demonstrated set back of DNAmAge. One small pilot study has been reported to have set back the DNAmAge clock over the course of 12 months by 1.5 (plus the one-year duration of the study) years in healthy men, using a combination of growth hormone, metformin, DHEA and two dietary supplements [29]. Two additional studies have demonstrated age reduction from diet and/or dietary supplement interventions. A subgroup of Polish women from the NU-AGE cohort suggested a reduction in biological age of 1.47 years after 1 year of a Mediterranean diet plus 400IU vitamin D3, and a 16-week trial using 4000IU of vitamin D3 in overweight or obese African Americans with suboptimal D status demonstrated a 1.85-year reduction in biological age [30, 31]. Herein we report comparable initial results based on diet and lifestyle interventions employed for eight weeks (preceded by a one-week education period).

## RESULTS

### DNA methylation clock

**Figure 1:**
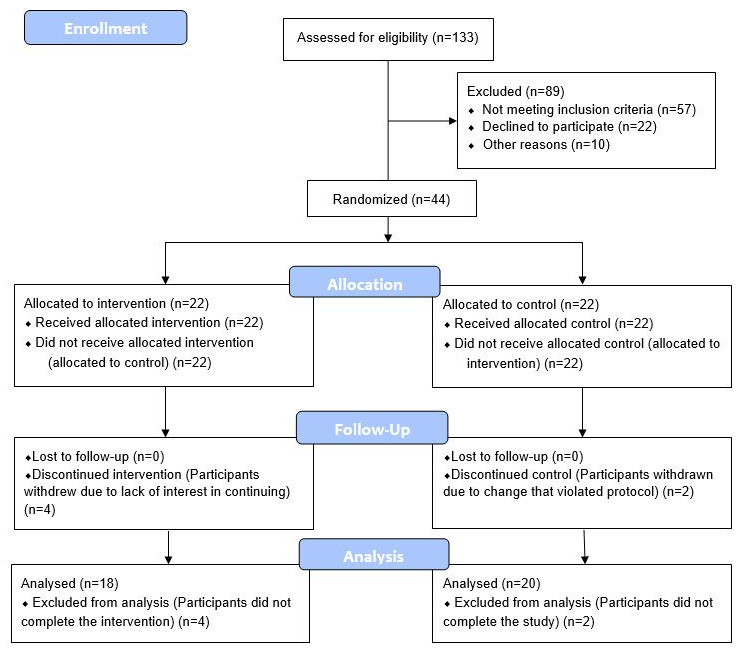
**CONSORT 2010 flow diagram.**

**Figure 2:**
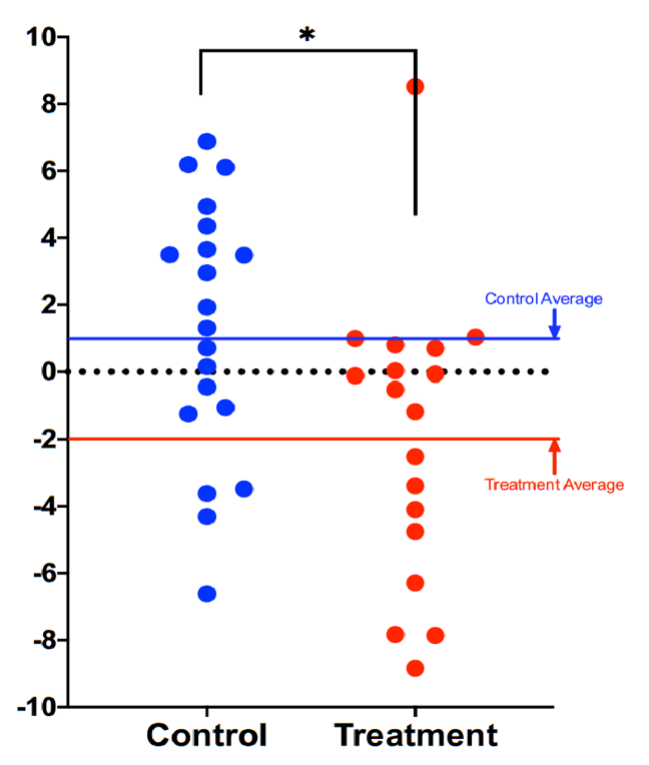
**Comparison of DNAmAge change between treatment and control groups.** Each dot is a subject, and the vertical axis represents difference in DNAmAge from the beginning to the end of the eight-week term. Participants scored an average 1.96 years younger, controls an average 1.27 years older. The age reduction of the treatment group strongly trended towards significance (p=0.066), while the age increase of the control group itself was not significant (p=0.153). The difference between control and treatment groups was significant at the level p=0.018 (unpaired two-tailed t-test). Long red and blue lines represent group averages (mean).

Compared to participants in the control group (n=20), participants in the treatment group scored an average 3.23 years younger at the end of the eight-week program according to the Horvath DNAmAge clock (p=0.018). Those in the treatment group (n=18) scored an average 1.96 years younger, at the end of the program compared to the same individuals at the beginning with a strong trend towards significance (p=0.066 for within group change). Control participants scored an average of 1.27 years older at the end of the study period, though this within-group increase was not statistically significant (p= 0.153). Comparison of DNAmAge change between treatment and control groups is shown in Figure 1 whereas within group changes for the treatment group are shown in Figure 2.

In both treatment and control groups, there was no net increase or decrease in methylation of the 353 sites that compose the Horvath clock. This finding suggests that the intervention did not lead to an overall increase in methylation of the Horvath clock sites, but rather it prompted a repositioning of clock’s CpG methylation patterns consistent with a younger biological age.

### Metabolic measures

In blood markers, the most significant change was a 25% decrease in mean triglycerides from 112 to 89 mg/dL (p=0.009) over the eight-week study period. As expected from a diet rich in folate, mean serum 5-methyltetrahydrofolate (5-MTHF) rose 15% from 78 to 88 nmol/L (p=0.004).

None of the other blood markers measured changed significantly compared to controls (glucose, hemoglobin A1C, total cholesterol, HDL cholesterol, LDL cholesterol, methionine, s-adenosylmethionine (SAM), s-adenosylhomocysteine (SAH) the ratio SAM:SAH and homocysteine), but within-group the participants in the treatment group showed significant decrease in total cholesterol (-22.8 mg/dL, p=0.004) and LDL cholesterol (-16.8 mg/dL, p=0.01).

### Emotional measures

Among the PROMIS markers of emotional health, there were no statistically significant changes between treatment and control groups after adjustment for baseline values. There was some trend towards reduced anxiety scores in the treatment group, however, these changes were not statistically significant.

## DISCUSSION

### Significance of results

The significance of these findings is multi-factorial, but primarily as the first demonstration of potential reversal of epigenetic age in a randomized, controlled clinical trial, accounting for any normal variability in epigenetic methylation. This is the second report of a diet and lifestyle intervention reducing biologic aging in individuals otherwise known to be healthy. Notably, the shorter timeframe of this study and the scale of potential reduction, while modest in magnitude, may correlate with meaningful socioeconomic benefits, and appears to have the potential to be broadly achievable.

Vitamin D3 at a dose of 4,000 IU/d for 16 weeks has previously been shown to decrease the DNAmAge clock measurement by 1.85 years in overweight/obese African Americans with a serum 25-hydroxyvitamin D [25(OH)d] <50 nmol/L [31]. Subsequently, a one-year regimen of daily injection of growth hormone plus one prescription drug and three nutritional supplements was shown to set back the DNAmAge clock by 1.5 years in 9 middle-aged men (plus the 1-year study duration = 2.5 years) [29]. More recently, a 1-year non controlled pilot trial involving 120 participants aged 65-79 years (including 60 Italians, 60 Poles) drawn from the larger NU-AGE cohort found a non-significant trend towards reversal of the DNAmAge clock after 1 year of a Mediterranean diet plus 400IU of vitamin D3 [30]. However, subgroup analysis did reveal a significant 1.47-year age decreases in female Polish participants (n=36) and in individuals with a baseline higher epigenetic age. It was noted in the study that Poland is a country with a non-Mediterranean baseline diet. In the present study, biological age set-back was achieved in eight weeks, using similarly non-invasive, and otherwise generally beneficial interventions known to have mechanistic plausibility for affecting methylation pathways.

The increase in circulating folate demonstrates that dietary sources and folate-producing probiotics can be an effective method of nutrient repletion. The reduction in serum triglycerides might be expected with a diet that lowered carbohydrate intake and glycemic response, plus exercise [32]. While we expected to see a decrease in homocysteine with an intervention that supplied additional dietary B vitamins and betaine, as well as exercise, the average starting homocysteine value of the treatment group was 10.9 umol/L, already within a range typically identified as “normal” (<15 umol/L).

### Effects of non-methyl donor factors on DNA methylation

The seminal work of Waterland and Jirtle in the Agouti mouse model marked a defining point in our understanding that nutrition elements could so affect DNA methylation marks as to silence gene expression and dramatically alter phenotype [33]. The power of nutrition to bring about transformative phenotypic changes has held up over the intervening years, most strongly in animal studies, but also in some limited human trials [33]. All of the aforementioned human trials (NU-AGE, TRIIM, Vitamin D3 study) and the present study were able to effect changes on the DNA methylome without extra-dietary supplementation of known methyl donor nutrients (e.g. folate, vitamin B12, choline, SAMe or betaine), supporting the concept of a far-reaching regulatory network on DNA methylation and representing a departure from previous studies that manipulated DNA methylation more directly with extra-dietary supplemental folate, B12 and other methyl donor nutrients [33–36].

The DNAmAge clock is computed from some sites that increase and others that decrease methylation with age, so a net increase in methylation is not the therapeutic target, compared to modifying methylation at appropriate sties. Since this study targeted a healthy methylation pattern, not limited to increased methylation, the prescribed diet contained TET demethylase-associated nutrients, such as vitamins A and C [13] and specific plant polyphenols such as curcumin and EGCG known to inhibit DNMT activity, in addition to high quantities of food-sourced methyl donor nutrients. As evidenced by younger DNAmAge (ie, “improved” methylation patterns) in the treatment group without overall increase (or decrease) in methylation, it suggests that these compounds collectively assist in elevating methylation and demethylation enzymatic support and thereby potentially regulate where methyl groups are applied and removed. In addition, the combination of polyphenols, rather than the use of singular phytonutrients, has been shown to deliver enhanced favorable effects on epigenetic changes [14, 37].

### Rationale for not using supplemental methyl donor nutrients

In designing the present study, extra-dietary supplementation of methyl donor nutrients was specifically avoided because a growing body of epidemiological evidence indicates potential long-term risks, to which the short-term studies were not sensitive. Although overall data are mixed, and certain conditions (e.g. pregnancy, macrocytic anemia, hyperhomocysteinemia, dietary limitations) often require extra-dietary supplementation, several trials have found a positive association between methyl donor supplementation and increased cancer risk: Published long-term follow up on 2,524 participants in the B-PROOF trial which assessed the effect of 2-3 years of daily supplementation with 400 mcg folic acid and 500 mcg vitamin B12 found an increased risk of overall cancer (HR 1.25, 95% CI 1.00-1.53), p=0.05) and colorectal cancer in particular (HR 1.77, 95% CI 1.08-2.90, p=0.02) [38]. A meta-analysis of 2 trials in Norway similarly reported that 800 mcg folic acid plus 400 mg vitamin B12 daily was associated with increased cancer outcomes and all-cause mortality [39]. In contrast, dietary folate intake from food was found to be inversely associated with non-muscle-invasive bladder cancer progression in a study that also found higher recurrence for folic acid intake [40], and baseline dietary folate intake was inversely associated with prostate cancer risk in a trial that subsequently identified an increased risk of prostate cancer in the treatment arm that received 1 mg folic acid per day for 10 years [41]. Also relevant is the demonstration, albeit in a small study, adding dietary supplements of folic acid, vitamin B6 and vitamin B12 to a vitamin D plus calcium intervention increased biological aging (sex-adjusted odds ratio 5.26 vs vitamin D plus calcium alone) during a 1-year intervention [42].

### Cautions and future directions

One significant limitation of this pilot trial is limited statistical power due to the relatively small sample size. Confirmation of these results is therefore needed in larger study groups and populations beyond middle-aged men.

It is not yet fully established whether interventions that slow any of the “methylation clocks” necessarily curtail risks of age-related disease. This unknown remains an important area of investigation by epidemiologists working to validate predictors of age-related morbidity and mortality, which would otherwise require very long clinical trials. The use of a multimodal intervention has advantages, as discussed above, however it also means it is not possible to attribute improved outcomes to any one element of the intervention. The combination of interventions used in this study may yet be improved upon and may be more impactful when further personalized. Future iterations of the intervention in continued clinical trials will attempt to optimize the program for efficacy, efficiency, scalability and affordability. An ever-evolving understanding of personalized application of such dietary and lifestyle interventions will likely lead to refinements to this kind of intervention that may further extend indicators of biological age.

Finally, it may be that emerging “omics” approaches continue to evolve our understanding of biological age prediction and reversal beyond DNA methylation alone [43]. Integration of our future understanding of multi-omics data should therefore be considered in the future trials of candidate age-delaying interventions.

## MATERIALS AND METHODS

### Study design and conduct

The trial design was approved by the Institutional Review Board of the National University of Natural Medicine (IRB number: RB100217) and registered at ClinicalTrials.gov (Identifier: NCT03472820). All trial procedures (visits, consent, randomization, and study visits) were conducted at the Helfgott Research Institute, 2220 SW 1^st^ Ave, Portland, OR 97201.

Participants were voluntarily recruited on a rolling basis from the general community surrounding Portland, OR, using flyers, online and newspaper advertising, and electronic newsletters posted at locations intended to attract more health-conscious individuals, such as gyms. Volunteers completed telephone pre-screening followed by an initial onsite screening visit including confirmation of eligibility, willingness to follow the study protocol, and informed consent. Full inclusion and exclusion criteria are provided as Supplementary Material.

**Figure 3:**
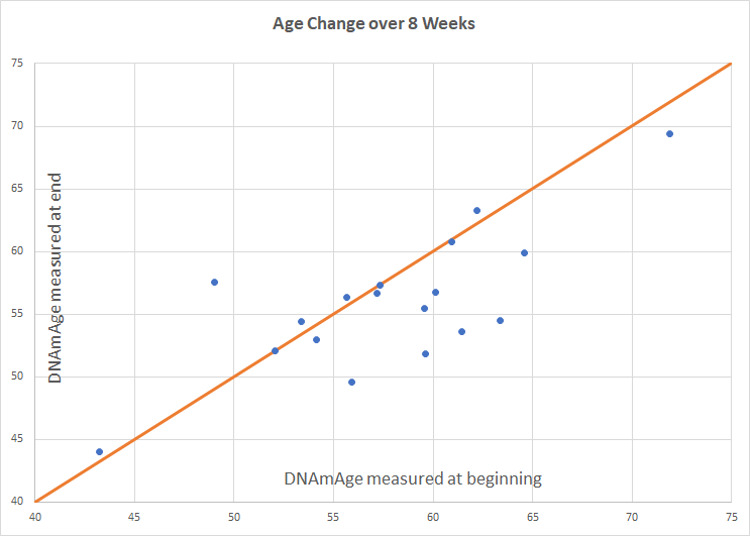
**Intervention group age change.** Participants scored an average of 1.96 years younger than baseline (p=0.066). Of 18 participants included in the final analysis, 8 scored age reduction, 9 were unchanged, and 1 increased in methylation age.

43 eligible adult males without history of recent or chronic disease, between the ages of 50-72, were recruited, consented, enrolled and randomized between March 2018 and August 2019. A CONSORT flow diagram is shown in Figure 3. Baseline characteristics are shown in Table 1. The age range of 50-72 was selected as a time when age-related vulnerabilities typically manifest, and the limitation to only male participants was to avoid the potential confounding factor of pre-, peri-, and post-menopausal sex hormone levels of the same age range in women.

**Table 1:** Baseline characteristics.

**Characteristics**	**Treatment group n = 21**			**Control group n = 22**	
	**Value**	**%**		**Value**	**%**
**Age, years (mean±SD)**	58.5 **±** 6.12			60.3 **±** 6.68	
**Race**					
Black or African American	0	0		2	9.1
American Indian or Native Alaskan	0	0		0	0
Native Hawaiian or Other Pacific Islander	0	0		0	0
Asian or Asian American	3	13.6		1	4.5
White, Caucasian or European American	18	81.8		18	81.8
Caribbean Islander or African National	0	0		0	0
More than one race	0	0		1	4.5
Unknown	0	0		0	0
**Education Level**					
Some high school	0	0		0	0
High school	0	0.00		3	0.14
Some university	1	0.05		1	0.05
2 year university	2	0.09		1	0.05
4 year university	6	0.27		4	0.18
Some graduate school	2	0.09		4	0.18
Graduate degree	11	0.50		9	0.41

A 3-week washout period (with written instruction) was initiated for all participants involving discontinuation of any nutrition supplements or herbal products not prescribed by a licensed healthcare provider for a medical condition. Allowable exceptions for dietary supplementation included low dose supplements, such as a common "1-a-day" multivitamin/mineral (i.e. high potency, high dose multivitamin/mineral products were not allowable), and/or other supplements taken for prevention: fish oil (up to 1 gram/day), vitamin D (up to 6000 IU/day), vitamin C (up to 1g/day), vitamin E (up to 400 IU/day). Notably, these supplements were not recommended or prescribed, but participants already taking these products were allowed to continue them during the trial, thus any effects would be captured in their baseline assessments. Participants agreed to avoid/discontinue any recreational drugs/substances, as well as to consume no alcohol, nicotine, marijuana or cannabinoids at least 1 week before scheduled study visits.

Study participants were randomized at the baseline visit according to a randomization sequence (randomization.com). Allocation concealment was accomplished by opening sealed, signed envelopes prepared by research staff not associated with the trial which were only opened at randomization.

Initial instructions, including a recorded instructional webinar and electronic technology webinar were provided at visit 1. To allow time for participant education to occur, participants in the treatment group were instructed to begin the 8-week intervention protocol (including dietary, supplement, and lifestyle changes) starting one week after the baseline visit. Saliva samples were collected at each of the three study visits (baseline, week 5 and week 9).

An overview of the intervention is provided in Table 2. Two nutritional supplements (PhytoGanix® and UltraFlora® Intensive Care, Metagenics Inc., 25 Enterprise Aliso Viejo, CA 92656 USA) were distributed at visits 1 and 2. Unused doses were collected, counted and recorded at visits 2 and 3. Dosing adherence was verified by the retrospective review of returned doses, direct queries about each component of the intervention during study visits, and by frequent communication with trial participants.

**Table 2:** Summary of dietary and lifestyle interventions*.

**Intervention category**	**Details**
Dietary Prescription	*Guidance per week:* **3 servings of liver** •(1 serving = 3 oz) •Preferably organic **5-10 eggs** •Ideally free-range, organic, omega-3 enriched *Guidance per day:* **2 cups of dark leafy greens** •Measured raw, chopped, and packed •Including kale, Swiss chard, collards, spinach, dandelion, mustard greens •Does not include salad greens such as romaine, iceberg, Spring mix **2 cups cruciferous vegetables** •Measured raw, chopped, and packed •Includes broccoli, cabbage, cauliflower, Brussels sprouts, bok choy, arugula, kale, mustard greens, watercress, rutabaga, kohlrabi, radish, Swiss chard, turnip **3 additional cups colorful vegetables** of your choosing (excluding white potatoes, sweetcorn) **1-2 medium beet** **4 tbsp (1/4 cup) pumpkin seeds** (or pumpkin seed butter) **4 tbsp (1/4 cup) sunflower seeds** (or sunflower seed butter) **1+ serving methylation adaptogens, choose from:** •1/2 cup berries (wild preferred) •1/2 tsp rosemary •1/2 tsp turmeric •2 medium cloves garlic •2 cups green tea (brewed 10 minutes) •3 cups oolong tea (brewed 10 minutes) **6 oz animal protein** •Grass-fed, pastured, organic and hormone/antibiotic-free **2 servings of low glycemic fruit** *General guidance:* **Organic** preferred over conventional **Stay hydrated** **Don’t eat between** 7pm and 7am **Include “healthy” oils** •Balance types of fat •E.g. coconut, olive, flaxseed and pumpkin seed oil **Avoid** added sugar/candy, dairy, grains, legumes/beans **Minimize** plastic food containers
Supplement Prescription	PhytoGanix®, a combination of organic vegetables, fruits, seeds, herbs, plant enzymes, prebiotics and probiotics at a dose of 2 servings daily, divided UltraFlora® Intensive Care, containing *Lactobacillus plantarum 299v* at a dose of 2 capsules daily, divided
Exercise Prescription	Minimum of 30 minutes of exercise per day for at least 5 days per week, at an intensity of 60-80% of maximum perceived exertion
Sleep Prescription	Average a minimum of 7 hours of sleep per night
Stress Management Prescription ^A^	Breathing exercise *Steps to Elicit the Relaxation Response* developed by Herbert Benson MD, twice daily

^A^Stress Management Recommendations were updated from the original Study Protocol as listed on ClinicalTrials.gov. All updates were IRB approved. *Patent pending.

Adherence to the program was supported by regular coaching sessions, delivered weekly during the first four weeks, and then at least every other week thereafter. Coaching sessions followed a pre-defined script that covered adherence to intervention guidelines and any changes to medications. A HIPAA-compliant electronic technology coaching tool was offered (MBody360, 640 Broadway 5A, New York, NY 10012) which contained reference instructions, meal planning ideas, optional recipes, and a shopping list. Participants could also use this tool to communicate with their assigned coach in between scheduled coaching sessions. Email, web platform and/or phone communication were other options for participants unable or unwilling to use MBody360.

### Determination of epigenetic age

Sample Handling: Saliva samples were stored at -70° C and remained frozen throughout the duration of the trial. Frozen samples were batch shipped overnight on dry ice to Yale University Center for Genome Analysis at the conclusion of clinical operations. Prior to shipment, sample IDs were assigned to plates such that each plate included a representative collection of samples from both allocation groups (treatment and control) and a distribution of samples from each trial visit, i.e. plates were not homogenous by group or by visit type, in an effort to equally distribute any random variability resulting from measurement across plates.

### DNA extraction

Oragene Saliva tubes were submitted and DNA extracted using the Perkin Elmer Chemagic 360 Instrument (kit# CMG-1081) following the manufacturer’s recommended protocol. An RNASE A digestion was added using 80μL of 4mg/μg Amercian Bioanalytical RNASE A (Part#AB12023-00100) after the 50 degree Celsius Oragene Saliva Incubation, before loading samples onto instrument.

### Genomic DNA and RNA

The quality of the RNA/DNA was evaluated by: A260/A280 and A260/A230 ratios (as supplied by the NanoDrop 1000 Spectrophotometer), both of which should be > 1.8. The gel electrophoresis pattern was consistent with non-degraded samples.

### Methods

Sample DNA was normalized to the recommended starting concentration, 1μg, for the Zymo EZ-96 DNA Methylation Kit (Cat No D5004). The samples underwent an overnight bisulfate conversion and were purified using the Zymo Methylation protocol. Samples proceeded directly to the Illumina Infinium HD assay (Illumina Methylation Epic Array Cat. No. WG-317-1001) to overnight whole genome amplification at 37 degrees Celsius. The following day samples were fragmented for 1hour at 37 degrees, precipitated at 4 degrees for 30 minutes, and pelleted at 4 degrees for 20 minutes at 3000xg. Samples were dried in a hood at room temperature for 1 hour and re-suspended in the recommended volume of RA1 following Illumina’s Infinium HD assay at 48 degrees Celsius for 1 hour. Samples were then denatured at 95 degrees Celsius for 20 minutes. Samples had a 10-minute cool down and then directly hybridized to Illumina Methylation Epic Array Cat. No. WG-317-1001. Sample placement in each chip was randomized. Hybridization was for 18 hours at 48 degrees Celsius using a stabilized hybridization oven. The following day arrays were washed and stained automatically using the Tecan Freedom Evo technology. Arrays were then be dipped in the UV protectant (Illumina’s XC3) for ten minutes and any excess removed. Arrays were dried for 1 hour in a vacuum desiccator. Arrays were scanned using Illumina’s iScan array scanner and raw files generated. Raw data files were imported into Illumina’s GenomeStudio software and a project created as well as QC parameters checked to ensure project went as expected. Scanned output files were analyzed and call rates calculated using Illumina’s GenomeStudio. The quality of the data was evaluated by both sample dependent and sample independent controls. Specifically, efficiency of target removal, non-specific binding, and appearance of cross-contamination was examined. All raw Data and the GenomeStudio project were uploaded to the password protected Keck Microarray Database.

### Data analysis

DNAmAge was calculated using the online Horvath clock available at https://dnamage.genetics.ucla.edu/. Analysis of epigenetic age was performed, blinded, on the final 18 participants in the treatment group and 20 participants in the control group. P values were computed as an unpaired 2-tailed t-test between the experimental group and control group, using the individual score differences (after treatment minus before) as a random variable.

### Data sharing

The data that support the findings will be available in Gene Expression Omnibus at https://www.ncbi.nlm.nih.gov/geo/, submission number GSE 149747, from 04-14-23 following an embargo from the date of publication to allow for commercialization of research findings.

## Supplementary Material

Supplementary Materials

## References

[1] Jin K, Simpkins JW, Ji X, Leis M, Stambler I. The critical need to promote research of aging and aging-related diseases to improve health and longevity of the elderly population. Aging Dis. 2014; 6:1–5. 10.14336/AD.2014.1210 25657847 PMC4306469

[2] Sen P, Shah PP, Nativio R, Berger SL. Epigenetic mechanisms of longevity and aging. Cell. 2016; 166:822–39. 10.1016/j.cell.2016.07.050 27518561 PMC5821249

[3] Kirkland JL, De Rooij SE, Goldman DP. Proof-of-concept clinical trials of interventions that target fundamental aging processes how can interventions be translated from the laboratory into clinical practice the economic returns to delayed aging: promise and pitfalls. Innov Aging. 2017 (Suppl 1); 1:1082. 10.1093/geroni/igx004.3967

[4] Li E, Zhang Y. DNA methylation in mammals. Cold Spring Harb Perspect Biol. 2014; 6:a019133. 10.1101/cshperspect.a019133 24789823 PMC3996472

[5] Horvath S, Raj K. DNA methylation-based biomarkers and the epigenetic clock theory of ageing. Nat Rev Genet. 2018; 19:371–84. 10.1038/s41576-018-0004-3 29643443

[6] Field AE, Robertson NA, Wang T, Havas A, Ideker T, Adams PD. DNA methylation clocks in aging: categories, causes, and consequences. Mol Cell. 2018; 71:882–95. 10.1016/j.molcel.2018.08.008 30241605 PMC6520108

[7] Johnson AA, Akman K, Calimport SR, Wuttke D, Stolzing A, de Magalhães JP. The role of DNA methylation in aging, rejuvenation, and age-related disease. Rejuvenation Res. 2012; 15:483–94. 10.1089/rej.2012.1324 23098078 PMC3482848

[8] Mitteldorf JJ. How does the body know how old it is? introducing the epigenetic clock hypothesis. Biochemistry (Mosc). 2013; 78:1048–53. 10.1134/S0006297913090113 24228927

[9] Rando TA, Chang HY. Aging, rejuvenation, and epigenetic reprogramming: resetting the aging clock. Cell. 2012; 148:46–57. 10.1016/j.cell.2012.01.003 22265401 PMC3336960

[10] Horvath S. DNA methylation age of human tissues and cell types. Genome Biol. 2013; 14:R115. 10.1186/gb-2013-14-10-r115 24138928 PMC4015143

[11] Langie SA, Moisse M, Declerck K, Koppen G, Godderis L, Vanden Berghe W, Drury S, De Boever P. Salivary DNA methylation profiling: aspects to consider for biomarker identification. Basic Clin Pharmacol Toxicol. 2017 (Suppl 3); 121:93–101. 10.1111/bcpt.12721 27901320 PMC5644718

[12] Quach A, Levine ME, Tanaka T, Lu AT, Chen BH, Ferrucci L, Ritz B, Bandinelli S, Neuhouser ML, Beasley JM, Snetselaar L, Wallace RB, Tsao PS, et al. Epigenetic clock analysis of diet, exercise, education, and lifestyle factors. Aging (Albany NY). 2017; 9:419–46. 10.18632/aging.101168 28198702 PMC5361673

[13] Hore TA. Modulating epigenetic memory through vitamins and TET: implications for regenerative medicine and cancer treatment. Epigenomics. 2017; 9:863–71. 10.2217/epi-2017-0021 28554227

[14] Arora I, Sharma M, Tollefsbol TO. Combinatorial epigenetics impact of polyphenols and phytochemicals in cancer prevention and therapy. Int J Mol Sci. 2019; 20:4567. 10.3390/ijms20184567 31540128 PMC6769666

[15] Sybesma W, Starrenburg M, Tijsseling L, Hoefnagel MH, Hugenholtz J. Effects of cultivation conditions on folate production by lactic acid bacteria. Appl Environ Microbiol. 2003; 69:4542–48. 10.1128/aem.69.8.4542-4548.2003 12902240 PMC169137

[16] Hariri M, Salehi R, Feizi A, Mirlohi M, Ghiasvand R, Habibi N. A randomized, double-blind, placebo-controlled, clinical trial on probiotic soy milk and soy milk: effects on epigenetics and oxidative stress in patients with type II diabetes. Genes Nutr. 2015; 10:52. 10.1007/s12263-015-0503-1 26577825 PMC4648806

[17] Ren H, Collins V, Clarke SJ, Han JS, Lam P, Clay F, Williamson LM, Andy Choo KH. Epigenetic changes in response to tai chi practice: a pilot investigation of DNA methylation marks. Evid Based Complement Alternat Med. 2012; 2012:841810. 10.1155/2012/841810 22719790 PMC3375016

[18] White AJ, Sandler DP, Bolick SC, Xu Z, Taylor JA, DeRoo LA. Recreational and household physical activity at different time points and DNA global methylation. Eur J Cancer. 2013; 49:2199–206. 10.1016/j.ejca.2013.02.013 23473616 PMC3686968

[19] e Silva Ade S, da Mota MP. Effects of physical activity and training programs on plasma homocysteine levels: a systematic review. Amino Acids. 2014; 46:1795–804. 10.1007/s00726-014-1741-z 24770903

[20] Spólnicka M, Pośpiech E, Adamczyk JG, Freire-Aradas A, Pepłońska B, Zbieć-Piekarska R, Makowska Ż, Pięta A, Lareu MV, Phillips C, Płoski R, Żekanowski C, Branicki W. Modified aging of elite athletes revealed by analysis of epigenetic age markers. Aging (Albany NY). 2018; 10:241–52. 10.18632/aging.101385 29466246 PMC5842850

[21] Pavanello S, Campisi M, Tona F, Lin CD, Iliceto S. Exploring epigenetic age in response to intensive relaxing training: a pilot study to slow down biological age. Int J Environ Res Public Health. 2019; 16:3074. 10.3390/ijerph16173074 31450859 PMC6747190

[22] Zannas AS, Arloth J, Carrillo-Roa T, Iurato S, Röh S, Ressler KJ, Nemeroff CB, Smith AK, Bradley B, Heim C, Menke A, Lange JF, Brückl T, et al. Lifetime stress accelerates epigenetic aging in an urban, African American cohort: relevance of glucocorticoid signaling. Genome Biol. 2015; 16:266. 10.1186/s13059-015-0828-5 26673150 PMC4699359

[23] Wolf EJ, Logue MW, Hayes JP, Sadeh N, Schichman SA, Stone A, Salat DH, Milberg W, McGlinchey R, Miller MW. Accelerated DNA methylation age: associations with PTSD and neural integrity. Psychoneuroendocrinology. 2016; 63:155–62. 10.1016/j.psyneuen.2015.09.020 26447678 PMC4695261

[24] Moore SR, McEwen LM, Quirt J, Morin A, Mah SM, Barr RG, Boyce WT, Kobor MS. Epigenetic correlates of neonatal contact in humans. Dev Psychopathol. 2017; 29:1517–38. 10.1017/S0954579417001213 29162165

[25] Watson NF, Badr MS, Belenky G, Bliwise DL, Buxton OM, Buysse D, Dinges DF, Gangwisch J, Grandner MA, Kushida C, Malhotra RK, Martin JL, Patel SR, et al, and Consensus Conference Panel. Joint consensus statement of the American academy of sleep medicine and sleep research society on the recommended amount of sleep for a healthy adult: methodology and discussion. J Clin Sleep Med. 2015; 11:931–52. 10.5664/jcsm.4950 26235159 PMC4513271

[26] Nilsson EK, Boström AE, Mwinyi J, Schiöth HB. Epigenomics of total acute sleep deprivation in relation to genome-wide DNA methylation profiles and RNA expression. OMICS. 2016; 20:334–42. 10.1089/omi.2016.0041 27310475 PMC4926204

[27] Carroll JE, Irwin MR, Levine M, Seeman TE, Absher D, Assimes T, Horvath S. Epigenetic aging and immune senescence in women with insomnia symptoms: findings from the women’s health initiative study. Biol Psychiatry. 2017; 81:136–44. 10.1016/j.biopsych.2016.07.008 27702440 PMC5536960

[28] Carskadon MA, Chappell KR, Barker DH, Hart AC, Dwyer K, Gredvig-Ardito C, Starr C, McGeary JE. A pilot prospective study of sleep patterns and DNA methylation-characterized epigenetic aging in young adults. BMC Res Notes. 2019; 12:583. 10.1186/s13104-019-4633-1 31526398 PMC6747743

[29] Fahy GM, Brooke RT, Watson JP, Good Z, Vasanawala SS, Maecker H, Leipold MD, Lin DT, Kobor MS, Horvath S. Reversal of epigenetic aging and immunosenescent trends in humans. Aging Cell. 2019; 18:e13028. 10.1111/acel.13028 31496122 PMC6826138

[30] Gensous N, Garagnani P, Santoro A, Giuliani C, Ostan R, Fabbri C, Milazzo M, Gentilini D, di Blasio AM, Pietruszka B, Madej D, Bialecka-Debek A, Brzozowska A, et al. One-year mediterranean diet promotes epigenetic rejuvenation with country- and sex-specific effects: a pilot study from the NU-AGE project. Geroscience. 2020; 42:687–701. 10.1007/s11357-019-00149-0 31981007 PMC7205853

[31] Chen L, Dong Y, Bhagatwala J, Raed A, Huang Y, Zhu H. Effects of vitamin D3 supplementation on epigenetic aging in overweight and obese African Americans with suboptimal vitamin D status: a randomized clinical trial. J Gerontol A Biol Sci Med Sci. 2019; 74:91–98. 10.1093/gerona/gly223 30256915 PMC6612014

[32] Hoyas I, Leon-Sanz M. Nutritional challenges in metabolic syndrome. J Clin Med. 2019; 8:1301. 10.3390/jcm8091301 31450565 PMC6780536

[33] Waterland RA, Jirtle RL. Transposable elements: targets for early nutritional effects on epigenetic gene regulation. Mol Cell Biol. 2003; 23:5293–300. 10.1128/mcb.23.15.5293-5300.2003 12861015 PMC165709

[34] Pauwels S, Ghosh M, Duca RC, Bekaert B, Freson K, Huybrechts I, Langie SA, Koppen G, Devlieger R, Godderis L. Maternal intake of methyl-group donors affects DNA methylation of metabolic genes in infants. Clin Epigenetics. 2017; 9:16. 10.1186/s13148-017-0321-y 28191262 PMC5297118

[35] Sae-Lee C, Corsi S, Barrow TM, Kuhnle GG, Bollati V, Mathers JC, Byun HM. Dietary intervention modifies DNA methylation age assessed by the epigenetic clock. Mol Nutr Food Res. 2018; 62:e1800092. 10.1002/mnfr.201800092 30350398

[36] Zhong J, Karlsson O, Wang G, Li J, Guo Y, Lin X, Zemplenyi M, Sanchez-Guerra M, Trevisi L, Urch B, Speck M, Liang L, Coull BA, et al. B vitamins attenuate the epigenetic effects of ambient fine particles in a pilot human intervention trial. Proc Natl Acad Sci USA. 2017; 114:3503–08. 10.1073/pnas.1618545114 28289216 PMC5380085

[37] Niedzwiecki A, Roomi MW, Kalinovsky T, Rath M. Anticancer efficacy of polyphenols and their combinations. Nutrients. 2016; 8:552. 10.3390/nu8090552 27618095 PMC5037537

[38] Oliai Araghi S, Kiefte-de Jong JC, van Dijk SC, Swart KM, van Laarhoven HW, van Schoor NM, de Groot LC, Lemmens V, Stricker BH, Uitterlinden AG, van der Velde N. Folic acid and vitamin B12 supplementation and the risk of cancer: long-term follow-up of the B vitamins for the prevention of osteoporotic fractures (B-PROOF) trial. Cancer Epidemiol Biomarkers Prev. 2019; 28:275–82. 10.1158/1055-9965.EPI-17-1198 30341095

[39] Ebbing M, Bønaa KH, Nygård O, Arnesen E, Ueland PM, Nordrehaug JE, Rasmussen K, Njølstad I, Refsum H, Nilsen DW, Tverdal A, Meyer K, Vollset SE. Cancer incidence and mortality after treatment with folic acid and vitamin B12. JAMA. 2009; 302:2119–26. 10.1001/jama.2009.1622 19920236

[40] Tu H, Dinney CP, Ye Y, Grossman HB, Lerner SP, Wu X. Is folic acid safe for non-muscle-invasive bladder cancer patients? an evidence-based cohort study. Am J Clin Nutr. 2018; 107:208–16. 10.1093/ajcn/nqx019 29529165 PMC6669327

[41] Figueiredo JC, Grau MV, Haile RW, Sandler RS, Summers RW, Bresalier RS, Burke CA, McKeown-Eyssen GE, Baron JA. Folic acid and risk of prostate cancer: results from a randomized clinical trial. J Natl Cancer Inst. 2009; 101:432–35. 10.1093/jnci/djp019 19276452 PMC2657096

[42] Obeid R, Hübner U, Bodis M, Graeber S, Geisel J. Effect of adding b-vitamins to vitamin D and calcium supplementation on CpG methylation of epigenetic aging markers. Nutr Metab Cardiovasc Dis. 2018; 28:411–17. 10.1016/j.numecd.2017.12.006 29395637

[43] Lorusso JS, Sviderskiy OA, Labunskyy VM. Emerging omics approaches in aging research. Antioxid Redox Signal. 2018; 29:985–1002. 10.1089/ars.2017.7163 28874057 PMC6104250

