# Correction for: Potential reversal of epigenetic age using a diet and lifestyle intervention: a pilot randomized clinical trial

**DOI:** 10.18632/aging.204197

**Published:** 2022-07-27

**Authors:** Kara N. Fitzgerald, Romilly Hodges, Douglas Hanes, Emily Stack, David Cheishvili, Moshe Szyf, Janine Henkel, Melissa W. Twedt, Despina Giannopoulou, Josette Herdell, Sally Logan, Ryan Bradley

**Affiliations:** 1 Institute for Functional Medicine, Federal Way, WA 98003,USA; 2 American Nutrition Association, Hinsdale, IL 60521, USA; 3 Helfgott Research Institute, National University of Natural Medicine, Portland, OR 97201, USA; 4 Helfgott Research Institute, National University of Natural Medicine, Portland, OR 97201, USA; 5 HKG Epitherapeutics (Hong Kong), Department of Molecular Biology, Ariel University, Israel, Gerald Bronfman Department of Oncology, McGill University, Montreal, Quebec, Canada; 6 Department of Pharmacology and Therapeutics, McGill University, Montreal, QC H3G 1Y6, Canada; 7 Helfgott Research Institute, National University of Natural Medicine, Portland, OR 97201, USA; 8 Division of Preventive Medicine, University of California, San Diego, CA 92023, USA

**This article has been corrected:** The authors corrected the **Data sharing** section. The correct text is presented below.

**Data sharing**

The data that support the findings will be available in Gene Expression Omnibus at https://www.ncbi.nlm.nih.gov/geo/ from 04-14-23 following an embargo from the date of publication to allow for commercialization of research findings.

