# Correction: Potential reversal of epigenetic age using a diet and lifestyle intervention: a pilot randomized clinical trial

**DOI:** 10.18632/aging.205700

**Published:** 2024-03-15

**Authors:** Kara N. Fitzgerald, Romilly Hodges, Douglas Hanes, Emily Stack, David Cheishvili, Moshe Szyf, Janine Henkel, Melissa W. Twedt, Despina Giannopoulou, Josette Herdell, Sally Logan, Ryan Bradley

**Affiliations:** 1 Institute for Functional Medicine, Federal Way, WA 98003, USA; 2 American Nutrition Association, Hinsdale, IL 60521, USA; 3 Helfgott Research Institute, National University of Natural Medicine, Portland, OR 97201, USA; 4 Helfgott Research Institute, National University of Natural Medicine, Portland, OR 97201, USA; 5 HKG Epitherapeutics (Hong Kong), Department of Molecular Biology, Ariel University, Israel, Gerald Bronfman Department of Oncology, McGill University, Montreal, Quebec, Canada; 6 Department of Pharmacology and Therapeutics, McGill University, Montreal, QC H3G 1Y6, Canada; 7 Helfgott Research Institute, National University of Natural Medicine, Portland, OR 97201, USA; 8 Division of Preventive Medicine, University of California, San Diego, CA 92023, USA

**Keywords:** DNA methylation, epigenetic, aging, lifestyle, biological clock

**This article has been corrected:** The authors wish to make the following corrections in the Abstract, Results, Figure 2, and Figure 3, to account for the participants’ chronological age increase (8+ weeks) at the end point evaluation compared to the baseline evaluation: “Those in the treatment group (*n* = 18) scored an average 2.04 years younger at the end of the program, measured by the Horvath DNAmAge clock, as compared to the same individuals at the beginning (*p* = 0.043 for within group change). Control participants scored an average of 1.10 years older at the end of the study period (*p* = 0.191).”

The comparison between test group and control group is not changed by this correction, since test and control groups are affected equally.

The corrected versions of the corresponding texts are provided below.

## ABSTRACT

**Manipulations to slow biological aging and extend healthspan are of interest given the societal and healthcare costs of our aging population. Herein we report on a randomized controlled clinical trial conducted among 43 healthy adult males between the ages of 50–72. The 8-week treatment program included diet, sleep, exercise and relaxation guidance, and supplemental probiotics and phytonutrients. The control group received no intervention. Genome-wide DNA methylation analysis was conducted on saliva samples using the Illumina Methylation Epic Array and DNAmAge was calculated using the online Horvath DNAmAge clock (2013). The diet and lifestyle treatment was associated with a 3.23 years decrease in DNAmAge compared with controls (*p* = 0.018). Those in the treatment group (*n* = 18) scored an average 2.04 years younger at the end of the program, measured by the Horvath DNAmAge clock, as compared to the same individuals at the beginning (*p* = 0.043 for within group change). Changes in blood biomarkers were significant for mean serum 5-methyltetrahydrofolate (+15%, *p* = 0.004) and mean triglycerides (−25%, *p* = 0.009). To our knowledge, this is the first randomized controlled study to suggest that specific diet and lifestyle interventions may reverse Horvath DNAmAge (2013) epigenetic aging in healthy adult males. Larger-scale and longer duration clinical trials are needed to confirm these findings, as well as investigation in other human populations.**

## RESULTS

### DNA methylation clock

Compared to participants in the control group (*n* = 20), participants in the treatment group scored an average 3.23 years younger at the end of the eight-week program according to the Horvath DNAmAge clock (*p* = 0.018). Those in the treatment group (*n* = 18) scored an average 2.04 years younger at the end of the program, measured by the Horvath DNAmAge clock, as compared to the same individuals at the beginning (*p* = 0.043 for within group change). Control participants scored an average of 1.10 years older at the end of the study period though this within-group increase was not statistically significant (*p* = 0.191). Comparison of DNAmAge change between treatment and control groups is shown in Figure 1 whereas within group changes for the treatment group are shown in Figure 2.

**Figure 2:**
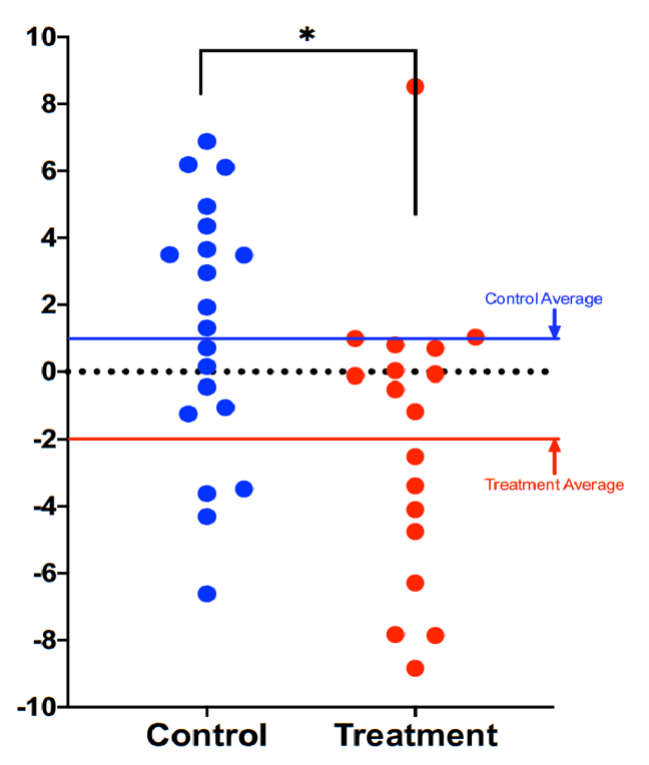
**Comparison of DNAmAge change between treatment and control groups.** Each dot is a subject, and the vertical axis represents difference in DNAmAge from the beginning to the end of the eight-week term. Those in the treatment group (*n* = 18) scored an average 2.04 years younger at the end of the program, measured by the Horvath DNAmAge clock, as compared to the same individuals at the beginning (*p* = 0.043 for within group change). Control participants scored an average of 1.10 years older at the end of the study period (*p* = 0.191). The difference between control and treatment groups was significant at the level *p* = 0.018 (unpaired two-tailed *t*-test). Long red and blue lines represent group averages (mean).

**Figure 3:**
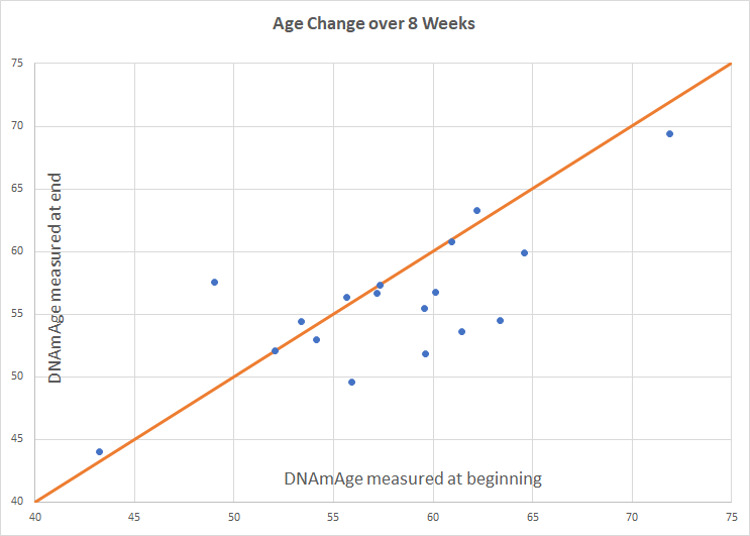
**Intervention group age change.** Participants in the treatment group (*n* = 18) scored an average 2.04 years younger at the end of the program, measured by the Horvath DNAmAge clock, as compared to the same individuals at the beginning (*p* = 0.043 for within group change). Of 18 participants included in the final analysis, 8 scored age reduction, 9 were unchanged, and 1 increased in methylation age.

