# Supplementary material for: Potential reversal of epigenetic age using a diet and lifestyle intervention: a pilot randomized clinical trial
Source: Aging (Albany NY). 2021 Apr 12;13(7):9419–32. doi: 10.18632/aging.202913 (PMC8064200; doi:10.18632/aging.202913)
Supplement: Supplementary Materials [file aging-13-202913-s001.pdf]

SUPPLEMENTARY MATERIALS

Supplementary information: Inclusion and exclusion criteria

ClinicalTrials.gov Identifier: NCT03472820.

Inclusion criteria

- Males, ages 50-72.
- Willing to adhere to 9 weeks of a dietary and lifestyle program including specific nutrition and exercise guidelines.
- Willing to avoid any over-the-counter medications, supplements or herbal products for the length of the study, except short-term use (<1 week) use at least 1 week before scheduled study visits.
- Willing to have blood drawn three times and abstain from food or beverage intake for 10-12 hours before blood draws.
- Willing to provide saliva samples.
- Willing to track food intake, sleep, stress management techniques, and exercise daily.
- Willing to drink a nutrient-enriched beverage and take a encapsulated probiotic daily.
- Willing and able to use electronic devices and connect to the internet.
- Able to speak, read and understand English.

Exclusion criteria

- Currently taking any of the following prescription medications.
  - Proton pump inhibitors: omeprazole (Prilosec, Prilosec OTC); aspirin and omeprazole (Yosprala); lansoprazole (Prevacid, Prevacid IV, Prevacid 24-Hour); ;dexlansoprazole (Dexilent, Dexilent Solutab); rabeprazole (Aciphex, Aciphex Sprinkle); pantoprazole (Protonix).
  - H2-blockers: nizatidine (Axid, Axid AR, Axid Pulvules); famotidine (Pepcid, Pepcid AC); cimetidine (Tagamet, Tagamet HB); ranitidine (Zantac).
  - These classes of medications are excluded due to direct (due to nutrient requirements for metabolism) and indirect (through impaired digestion and assimilation of nutrients).
- Use of nutrition supplements or herbal products prescribed by a licensed healthcare provider for a medical condition.
- Currently following a prescribed dietary/ lifestyle program or initiate within the 30 days prior to baseline.
- Initiation of or changes to an exercise regimen within 30 days prior to baseline.
- Use of nicotine, marijuana or cannabinoids (including CBD products) or recreational drugs/substances (such as but not limited to cocaine, phencyclidine [PCP], and methamphetamine) current/within the last 30 days or use during the study.
- Have a diagnosis of cardiovascular disease, kidney disease, liver disease, diabetes, autoimmune disease, high blood pressure, or cancer *(does not include basal cell carcinoma, squamous cell carcinoma, and/or carcinoma in situ of the cervix).*
- Have a diagnosis of an immunodeficiency condition, such as Human Immunodeficiency Virus (HIV) infection or Acquired Immune Deficiency Syndrome (AIDS).
- Have a diagnosis of neurodegenerative conditions such as, amyotrophic lateral sclerosis (ALS), Parkinson’s disease, Multiple Sclerosis, or Alzheimer’s disease.
- Excessive alcohol consumption (more than 4 drinks per day or 14 per week on average).
- Known sensitivity, intolerance or allergy to ingredients in the study supplements or in the recommended dietary therapy.
- Currently receiving intravenous nutrient therapy.
- Currently participating in another interventional research study or participated in another interventional study within the last 3 weeks prior to baseline.
